# 三通道气相色谱法监测大气中CH_4_、CO、CO_2_、N_2_O和SF_6_

**DOI:** 10.3724/SP.J.1123.2022.02011

**Published:** 2022-08-08

**Authors:** Haixiang HONG, Kunpeng ZANG, Yuanyuan CHEN, Yi LIN, Jiaxin LI, Xuemei QING, Shanshan QIU, Haoyu XIONG, Kai JIANG, Shuangxi FANG

**Affiliations:** 1.浙江工业大学环境学院, 浙江 杭州 310014; 1. College of Environment, Zhejiang University of Technology, Hangzhou 310014, China; 2.浙江工业大学, 浙江碳中和创新研究院, 浙江 杭州 310014; 2. Institute of Zhejiang Carbon Neutral Innovation, Zhejiang University of Technology, Hangzhou 310014, China

**Keywords:** 气相色谱法, 温室气体, 在线监测, 碳中和, gas chromatography (GC), greenhouse gases, on-line monitoring, carbon neutral

## Abstract

我国正处于“碳达峰、碳中和”的关键时期,准确认识我国温室气体浓度时空格局以及变化对于评估“碳达峰”和“碳中和”行动成效非常重要。当前我国近地面温室气体高精度监测主要依赖进口的光学监测主机,单台仪器成本高且监测要素有限。为此,该研究基于传统的气相色谱法,自主设计了一套三通道气相色谱分析系统,在单台仪器上实现了5种主要长寿命温室气体(CH_4_、CO、CO_2_、N_2_O和SF_6_)的高精度监测。对该系统的精密度、线性响应情况和准确度进行的针对性测试实验表明系统检测性能满足世界气象组织/全球大气观测(WMO/GAW)质控标准。针对环境浓度的CH_4_、CO、CO_2_、N_2_O和SF_6_的连续分析精密度分别达0.08%、1.90%、0.05%、0.08%、0.66%。准确度测试中,5种气体(CH_4_、CO、CO_2_、N_2_O和SF_6_)使用回归方程计算所得值与标称摩尔分数间的偏差分别达0.15×10^-9^、0.20×10^-9^、0.37×10^-6^、0.35×10^-9^、0.02×10^-12^(摩尔分数),CH_4_、CO、CO_2_、N_2_O和SF_6_仪器响应值与标称摩尔分数的线性拟合相关系数(*R*^2^)均为0.9999,线性拟合残差和准确度基本达到WMO/GAW拓展质控目标。该系统对杭州城区大气温室气体在线连续监测结果显示,2021年5~7月期间大气CH_4_、CO、CO_2_和N_2_O呈明显的日变化特征,主要受人为活动影响。综合测试和试运行结果表明,该研发系统具备良好的精密度、准确度、线性和稳定性,与目前国内广泛进口的仪器相比,具有技术自主可控、运行成本更低、自动化水平更高等优势,能满足多种温室气体在线监测研究的需求。

## Abstract (en)

China is approaching a critical period of carbon peak and carbon neutrality. To assess the impact of carbon peak and carbon neutrality measures, an accurate understanding of the variations of the spatial and temporal distribution of greenhouse gases is crucial. Gas chromatography, a classical approach for greenhouse gas observation, can be employed for the high-precision analysis of partial greenhouse gases. In this research, a new greenhouse gas analytical system capable of measuring five gases (CH_4_, CO, CO_2_, N_2_O and SF_6_) on a single instrument was developed based on the traditional gas chromatography approach. The following are the chromatographic operation conditions. The carrier gases were high purity N_2_(99.999%) and argon-methane (5% methane in argon, 99.9999%), and a stainless steel switching valve triggered the injection. Compressed CH_4_, CO, CO_2_, N_2_O and SF_6_ mixed standard gases were stored in a 0.029 m^3^ aluminum alloy steel cylinder for this experiment. After numerous rounds of calibration by Greenhouse Gas Laboratory of Atmospheric Sounding Center of China Meteorological Administration, the gas scale met the primary standard of World Meteorological Organization (WMO). The main performance of the system, including the measurement precision, accuracy and linear response, was tested. The results showed that the detection performance of the system met the quality standards of WMO/Global Atmospheric Watch (GAW). Precision test results indicated that the relative standard deviations (RSDs) of the mole fractions of CH_4_, CO, CO_2_, N_2_O and SF_6_ were 0.08%, 1.90%, 0.05%, 0.08%, and 0.66%, respectively. For the linear and accuracy test, the C1-C5 tested standard gases were employed and the deviations of five gases (CH_4_, CO, CO_2_, N_2_O and SF_6_) between the calculated mole fractions of the regression equation and calibrated mole fractions were 0.15×10^-9^, 0.20×10^-9^, 0.37×10^-6^, 0.35×10^-9^ and 0.02×10^-12^, respectively. For CH_4_, CO, CO_2_, N_2_O and SF_6_, the linear regression coefficients (*R*^2^) between the peak areas or heights and calibrated mole fractions were 0.9999. The linear regression residual and accuracy could roughly meet the expanded target of WMO/GAW quality control. The atmospheric greenhouse gases in the Hangzhou urban area were continuously measured from May 2021 to July 2021 using the developed system. The results revealed that atmospheric CH_4_, CO, CO_2_ and N_2_O have visible diurnal variation characteristics that were primarily affected by anthropogenic emissions. The target standard gases were measured every 2 h to monitor the stability of the system operation, and the gas mole fractions of the system response were routinely computed and compared with the assigned calibrated values. The results demonstrated that the system had good stability during the observation period and could meet the requirements of high-precision monitoring. The comprehensive test and trial operation results showed that the developed system had good precision, accuracy, linearity and stability.

全球气候变化已成为人类最迫切需要解决的环境问题。世界气象组织(World Meteorological Organization, WMO)指出,2020年全球平均气温较工业化前期(1750年)已经上升了(1.2±0.1) ℃^[1]^。若不施加人为干预,到21世纪末全球平均温度将远超《巴黎协定》的较工业化前期升温幅度控制在1.5~2 ℃的目标。化石燃料燃烧、工业和农业生产等人为活动排放的温室气体是全球气候变化的重要诱因。1997年,《京都议定书》将CH_4_、CO_2_、N_2_O、SF_6_、氢氟碳化物(HFCs)和全氟碳化物(PFCs)规定为强制减排的温室气体。CO_2_是空气中除水汽外含量最多的温室气体,对大气辐射强迫的贡献约占所有长寿命温室气体的66%,其全球人为来源主要是化石燃料的燃烧和水泥生产^[2]^。CH_4_、N_2_O和SF_6_也是重要的微量温室气体,其源汇和排放通量的时空变化具有很大的不确定性^[3,4]^。CO是一种间接温室气体,其辐射强迫约为CO_2_的两倍,全球65%的CO来源于人为排放,因其在对流层与OH自由基反应进而影响CH_4_的寿命而逐渐被重视^[5,6]^。

全球温室气体正以近30年来最快速度增长,世界气象组织第17期温室气体公报(World Meteorological Organization Greenhouse Gases Bulletin, 2021)指出:2020年全球大气中CO_2_、CH_4_和N_2_O的平均摩尔分数分别为413.2×10^-6^±0.2×10^-6^、1889×10^-9^±2×10^-9^和333.2×10^-9^±0.1×10^-9^,分别比前一年增加了2.7×10^-6^±0.3×10^-6^、12×10^-9^±3×10^-9^和1.2×10^-9^±0.1×10^-9^。温室气体观测是国内外开展气候变化研究的基本形式之一,观测全球大气温室气体混合比对于开展长期气候监测以及评估全球尺度温室气体通量具有重大意义^[7]^。

我国温室气体监测整体起步较晚,近年虽然温室气体高精度监测网络有所扩大,但远不能满足区域或国家尺度碳源汇高精度评估的需要。当前我国温室气体高精度监测仪器主要采用基于波长扫描光腔衰荡光谱(WS-CRDS)或者离轴积分腔光谱监测系统(ICOS),但其完全来自于美国进口,且单台仪器测定要素有限,成本较高,返厂维修时间长。气相色谱法作为经典的温室气体分析方法,可用于CH_4_、CO_2_等温室气体的高精度分析,但是在科学文献中很少有关于同时分析多种主要长寿命温室气体的研究^[8]^。王跃思等^[9]^通过对气相色谱仪进样、分析气路和阀驱动系统的改造,可以同时测定陆地生态系统CO_2_、CH_4_和N_2_O的通量,仪器的灵敏度、分辨率和精密度均较高;张丽萍等^[10]^通过选配特殊色谱柱,并优化气路设置方案,开发了可同时测定大气中CH_4_、CO_2_、CO及其他烃类的气相色谱系统,该系统具有响应时间短、重现性和灵敏度高的特点;Pascale等^[11]^开发了气相色谱仪搭配介质阻挡放电等离子体检测器的方法,可同时检测CO_2_和N_2_O,仪器显示出良好的选择性、线性和准确度。2010年方双喜等^[12]^基于传统气相色谱法,自主设计并组装调试了一套双通道气相色谱分析系统,可同时在线连续监测大气中CH_4_、CO、N_2_O和SF_6_的摩尔分数,精度、重现性和灵敏度可较好地满足世界气象组织全球大气观测网的质控指标。

为进一步优化气相色谱法,降低运行成本,实现主要长寿命温室气体的全要素监测,提高自动化智能化监测水平,本研究基于传统气相色谱仪,自主设计集成一套改进型三通道气相色谱,实现CH_4_、CO_2_、CO、N_2_O和SF_6_的高精度监测,并通过对杭州市区大气温室气体开展试监测对仪器性能指标深入评估。

## 1 实验部分

### 1.1 仪器与试剂

预装Agilent Chemstation色谱工作站的气相色谱仪(Agilent 7890B, Agilent Inc.,美国)、氢火焰离子化检测器(flame ionization detector, FID)、微池电子捕获检测器(micro-electrical capture detector, μ-ECD)、镍转化炉(CAT)、4通双位切换阀、6通双位切换阀、10通双位切换阀、16位样品选择阀(如图1所示)、质量流量计(HORIBA STEC, MT-51)、电磁阀、过滤器(Whatman, 2702t)、NITTA采样管、氢气发生器、零空气发生器、0.5 nm分子筛不锈钢填充柱、Unibeads不锈钢填充柱、HayeSep Q不锈钢填充柱、高纯N_2_载气(纯度99.999%)、氩甲烷载气(5%甲烷在氩气中,纯度99.9999%)等。

**图1:**
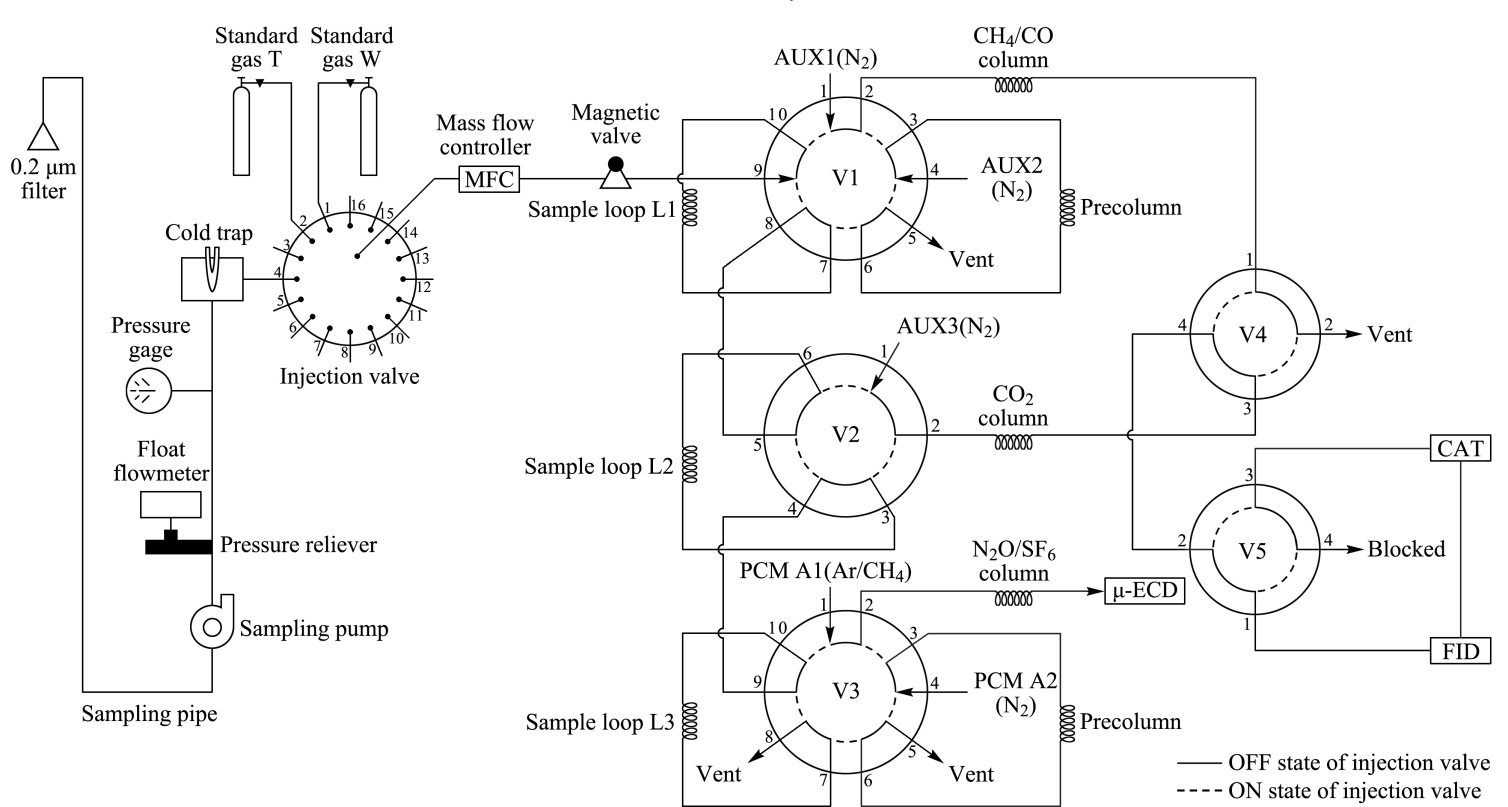
三通道气相色谱系统示意图

购自世界气象组织的实验测试用标准气体均为以清洁空气为底气的CH_4_、CO、CO_2_、N_2_O、SF_6_混合标准气体,存储于0.029 m^3^铝合金钢瓶(Scott-Marrin Co.,美国)中,其摩尔分数见表1。该标气序列经中国气象局大气探测中心温室气体实验室采用一级标准气体多轮标校,可溯源至WMO一级标准(primary standard)。

**表1:** 系统测试标准气体的摩尔分数

Standard gas	Numbered	CH_4_/10^-9^	CO/10^-9^	CO_2_/10^-6^	N_2_O/10^-9^	SF_6_/10^-12^
Tested gas	C1	1922.6	146.8	374.94	304.59	8.45
	C2	2047.6	229.4	408.88	313.27	9.21
	C3	2236.8	255.8	453.38	332.69	10.86
	C4	2431.3	355.3	497.89	345.84	12.61
	C5	2622.3	428.9	595.47	349.06	14.16
	C^*^	2005.9	191.6	422.25	334.37	10.76
Working gas	W	2005.9	191.6	422.25	334.37	10.76
Target gas	T	2075.4	185.6	414.33	322.27	10.12

### 1.2 监测方法

为评估该仪器性能,在浙江工业大学朝晖校区楼顶对城市大气温室气体浓度开展初步监测。如图1所示,进样口距地面20 m,安装0.2 μm过滤器以去除颗粒物,空气经过滤器和采样管,由采样泵抽至实验室。泵之后配置压力释放控制器,保证输出压力在103.4 kPa左右,气路中安装浮子流量计和压力计以监视气路压力变化,进气压力控制在68.95~103.4 kPa。空气进入色谱阀箱之前经超低温冷阱(-50 ℃)去除大部分水分,进样流量设置为250 mL/min。

三通道色谱系统如图1所示,样气由16位样品选择阀控制依次进入定量环Loop 1、Loop 2、Loop 3,冲洗并充满定量环后,完成系统进样,进样时间为0.5 min。然后,各定量环内样气经切换阀切换,分3通道进入检测器。该系统阀的切换、载气及反吹气压力等关键参数均由工作站控制,可实现3个通道自动化进样和程序化测定。三通道测定过程如下。

CH_4_/CO通道:0.75 min时V1、V4切换为ON状态,V5为OFF状态,AUX 1(辅助压力控制)载气将Loop 1内样气带入预柱和主柱,依次完成CH_4_和CO的分离。CH_4_首先被载气带出主柱,直接进入FID测定。CH_4_出峰后,1.8 min时V5切换为ON状态,CO经镍转化炉(CAT)被镍催化剂在高温(385 ℃)下转化成CH_4_,再进入FID测定。最后,5 min时V1切换为OFF状态,AUX 2载气将重碳氢化合物等后流出组分从预柱中反向吹出,避免其对下一个样品的分析产生干扰。

CO_2_通道:2.9 min时V2切换为ON状态,3.8 min时V4切换为OFF状态。AUX 3载气将Loop 2内样气带入HayeSep Q填充柱完成CO_2_的分离。CO_2_经CAT被镍催化剂在高温(385 ℃)下转化成CH_4_,再进入FID测定。

N_2_O/SF_6_通道:0.75 min时V3切换为ON状态,PCM A1载气将Loop 3内样气带入预柱和主柱,完成N_2_O和SF_6_的分离,再依次进入μ-ECD测定。N_2_O、SF_6_出峰后,8.5 min时V3切换成OFF状态,PCM A2载气将预柱中的后流出组分反向吹出。图2是由该软件采集的CH_4_、CO、CO_2_、N_2_O和SF_6_典型的色谱图。

**图2:**
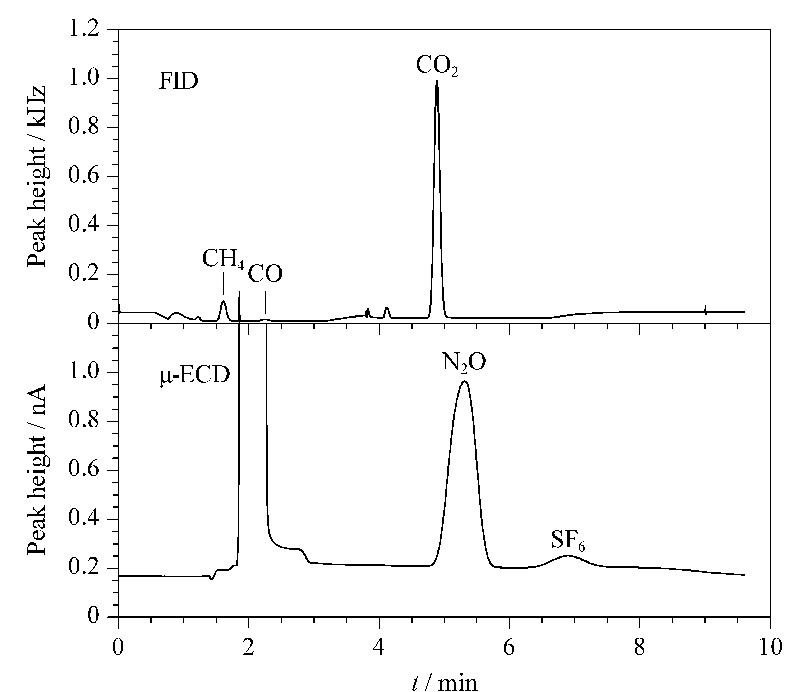
CH_4_、CO、CO_2_、N_2_O和SF_6_的典型色谱图

试运行监测期间,所用两瓶混合标准气体分别为工作气W和目标气T,在线监测的这5种气体的测定相邻时间间隔均为20 min(空气/工作气交替分析)。为监控系统运行的稳定性,每2 h测定目标气T,计算系统响应的温室气体摩尔分数,与给定的标称摩尔分数比对分析。

### 1.3 色谱条件

色谱条件如表2所示。载气为高纯N_2_和氩甲烷,进样方式为阀进样,进样体积为30 mL,进样流量为250 mL/min,柱温为65 ℃,分析时间为9.5 min。FID温度为175 ℃,氢气流速为75 mL/min,空气流速为280 mL/min,镍转化炉温度为385 ℃, μ-ECD温度为390 ℃。

**表2:** 色谱工作条件

Channel	Detected gas	Quantitative loop/mL	Precolumn			Analytical column			Carrier gas			
			Specification	Packing		Specification	Packing		Species		Pressure/kPa	
1	CH_4_/CO	10	1.2 m, stainless steel	60-80 meshes molecular sieves		1.2 m, stainless steel	60-80 meshes Unibeads 1 S			high purity N_2_ (99.999%)		103.43 (backflush gas: 137.9)
2	CO_2_	5	1.2 m, stainless steel	60-80 meshes molecular sieves		4.5 m, stainless steel	80-100 meshes HayeSep Q			high purity N_2_ (99.999%)		137.9
3	N_2_O/SF_6_	15	2 m, stainless steel	80-100 meshes HayeSep Q		2 m, stainless steel	80-100 meshes HayeSep Q			high purity N_2_ (99.999%) and argon-methane (99.9999%)		68.95 (backflush gas: 68.95)

## 2 结果与讨论

精密度、线性和准确度是仪器和分析方法测试验证的重要参数,为验证三通道气相色谱系统及方法是否满足大气温室气体监测需求,本研究对系统精密度、线性性能和准确度进行了针对性实验测试,同时将数据与世界气象组织/全球大气观测(WMO/GAW)实验室间比对分析的质控标准进行了比对。

### 2.1 精密度测试

在试验研究中,分析方法的精密度是指在规定的条件下,从同一均匀样品的多次抽样中获得的一系列测量值之间的一致性的密切程度^[13]^。精密度是评估气相色谱系统性能的重要指标,三通道气相色谱系统通过计算重复进样的相对标准偏差(RSD)来评价精密度。将标准气体C^*^接入系统,并连续进样分析97次。CH_4_、CO和SF_6_采用峰高定量,N_2_O和CO_2_采用峰面积定量^[12]^,利用测量的前后两个标准气体响应信号(峰高或峰面积)平均值和标称摩尔分数计算该次测量的摩尔分数^[12]^,计算的摩尔分数的标准偏差(SD)和RSD如表3所示。CH_4_、CO、CO_2_、N_2_O和SF_6_的RSD分别为0.08%、1.90%、0.05%、0.08%和0.66%,SD分别为1.70×10^-9^、3.63×10^-9^、0.20×10^-6^、0.26×10^-9^、0.07×10^-12^。由表4可知,与青海瓦里关站原有的双通道气相色谱^[12]^相比, SF_6_的标准偏差降低了0.03×10^-12^。卿雪梅等^[14]^利用Picarro G-2401型分析仪分析CO_2_和CH_4_的重复性标准偏差为0.02×10^-6^和0.1×10^-9^,该系统与Picarro G-2401型分析仪测定的精密度尚存在一定差距。但CH_4_、CO、CO_2_、N_2_O均能达到WMO/GAW实验室间比对分析的拓展目标(CH_4_:±4×10^-9^; CO:±5×10^-9^; CO_2_:±0.2×10^-6^; N_2_O:±0.3×10^-9^),系统的精密度表现良好。

**表3:** 利用峰高或峰面积定量计算目标物得到的 摩尔分数及其偏差和RSD(*n*=95)

Analyte	Average mole fraction	SD(1σ)	RSD/%
CH_4_	2005.0×10^-9^	1.70×10^-9^	0.08
CO	191.6×10^-9^	3.63×10^-9^	1.90
CO_2_	422.24×10^-6^	0.20×10^-6^	0.05
N_2_O	334.39×10^-9^	0.26×10^-9^	0.08
SF_6_	10.76×10^-12^	0.07×10^-12^	0.66

CH_4_, CO, and SF_6_ are quantified by peak height; N_2_O and CO_2_ are quantified by peak area.

**表4:** 色谱系统精密度比较

Analyte	SDs					RSDs/%			
	Ref. [9]		Ref. [12]	This work		Ref. [10]	Ref. [11]	Ref. [12]	This work
CH_4_	32×	10^-9^	0.63×10^-9^	1.70×10^-9^		0.5-3.5	-	0.04	0.08
CO	-		0.55×10^-9^	3.63×10^-9^		0.5-3.5	-	0.50	1.90
CO_2_	1.29×	10^-6^	-	0.20×10^-6^		0.5-3.5	0.4-6.6	-	0.05
N_2_O	5×	10^-9^	0.25×10^-9^	0.26×10^-9^		-	1.0-5.1	0.08	0.08
SF_6_	-		0.10×10^-12^	0.07×10^-12^		-	-	1.80	0.66

-: unmeasured.

### 2.2 线性测试

将C1、C2、C3、C4和C5共5瓶标准气体接入气相色谱系统,依次重复进样测定55次,共计275个响应结果。CO_2_和N_2_O在仪器上的峰面积响应值标准偏差较大,且FID为线性响应,而ECD一般为非线性响应^[12]^,故利用最小二乘法,对CH_4_、CO和SF_6_峰高平均值(*y*)与标称摩尔分数(*x*)进行线性拟合,对N_2_O、CO_2_峰面积平均值(*y*)与标称摩尔分数(*x*)进行二次多项式拟合。

由表5可知,CH_4_、CO、CO_2_、N_2_O和SF_6_的响应值与标称摩尔分数的线性相关系数(*R*^2^)均为0.9999,表明系统对摩尔分数范围分别为1922.6×10^-9^~2622.3×10^-9^的CH_4_、146.8×10^-9^~428.9×10^-9^的CO、374.94×10^-6^~595.47×10^-6^的CO_2_、304.59×10^-9^~349.06×10^-9^的N_2_O和8.45×10^-12^~14.16×10^-12^的SF_6_有良好的线性响应。

**表5:** 仪器线性响应结果

Analyte	Regression equation	R^2^	Mole fraction range
CH_4_	y=0.023x-0.082	0.9999	1922.6×10^-9^-2622.3×10^-9^
CO	y=0.007x-0.148	0.9999	146.8×10^-9^-428.9×10^-9^
CO_2_	y=-0.002x^2^+41.295x-343.036	0.9999	374.94×10^-6^-595.47×10^-6^
N_2_O	y=0.006x^2^+22.861x-1816.137	0.9999	304.59×10^-9^-349.06×10^-9^
SF_6_	y=1.992x+1.083	0.9999	8.45×10^-12^-14.16×10^-12^

y: peak height (CH4, CO, SF6) or peak area (CO2, N2O); x: mole fraction.

在线性回归分析中,与分析方法相关的不确定性的估计在数据质量验证中尤为重要,是环境分析的重要组成部分。测量值的可变性可能是与不准确或缺乏高精度有关,其综合了与分析程序相关的所有误差源的影响^[15,16]^。为验证线性回归模型的假设可行性,利用误差的估计值即残差^[17]^进一步分析线性特征,结果如图3。利用一次线性模型拟合的CH_4_、CO和SF_6_残差平均值分别为±0.4×10^-9^、±1.2×10^-9^、±0.01×10^-12^, N_2_O、CO_2_利用二次多项式拟合模型的残差平均值分别为±0.11×10^-9^、±0.23×10^-6^, CH_4_、CO、N_2_O和SF_6_均能达到WMO/GAW实验室间比对分析拓展目标(CH_4_:±4×10^-9^; CO:±5×10^-9^; N_2_O:±0.3×10^-9^; SF_6_:±0.05×10^-12^)^[18,19]^,达到WMO/GAW大气观测结果比对要求。

**图3:**
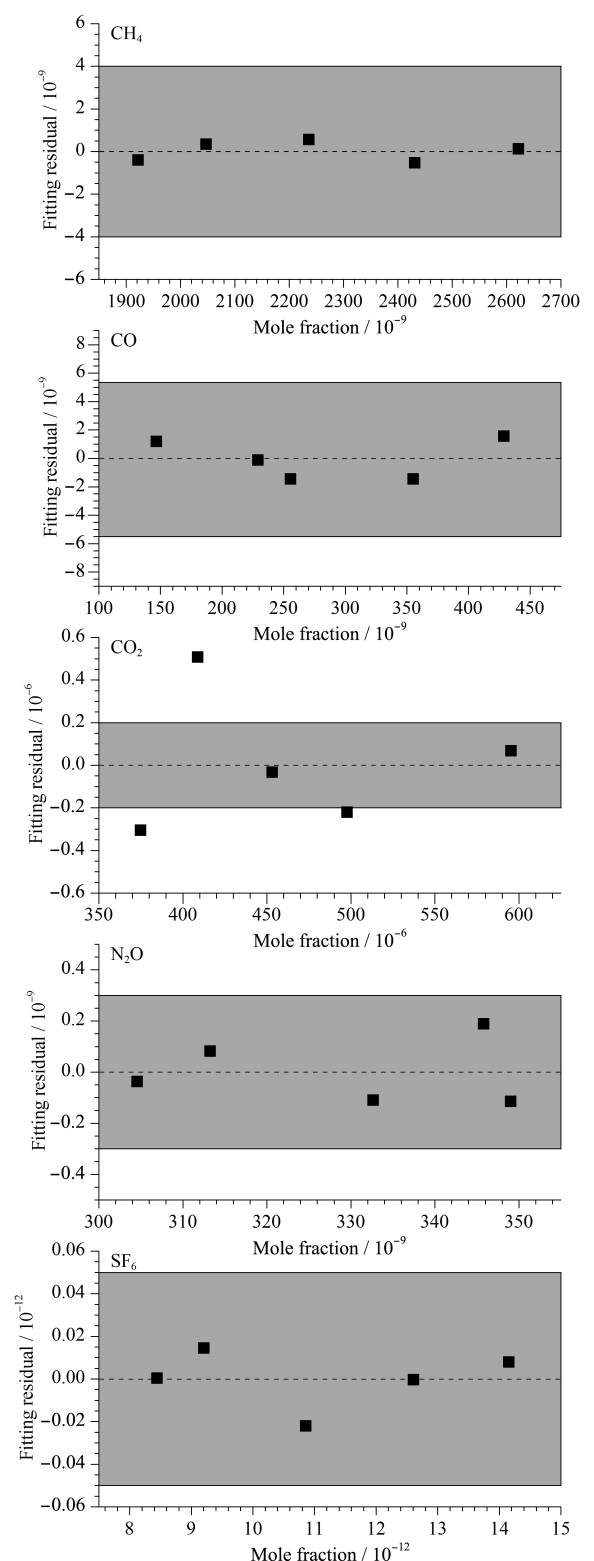
标准气体拟合残差分布

### 2.3 准确度测试

除了评估精密度以外,准确度测试也是评估仪器性能的重要指标之一。准确度是指测定值和常规真值或可接受的参考值之间的接近程度,与测量偏差有关^[20]^。

选择浓度较为接近环境大气浓度的工作标准气体能够提高N_2_O观测精度^[21]^,故测试准确度时选择最接近环境浓度的C3当作未标定气体。利用回归模型,将C1、C2、C4、C5 4瓶标准气体CH_4_、CO和SF_6_峰高平均值(*y*)与标称摩尔分数(*x*)进行线性拟合,N_2_O、CO_2_峰面积平均值(*y*)与标称摩尔分数(*x*)进行二次多项式拟合。建立方程,然后将C3当作未标定空气,利用C3标准气体进样响应值和回归方程,计算摩尔分数,并与其标称摩尔分数比对,结果见表6。利用回归方程计算的CH_4_、CO、CO_2_、N_2_O和SF_6_值与标称摩尔分数偏差分别为0.15×10^-9^、0.20×10^-9^、0.37×10^-6^、0.35×10^-9^和0.02×10^-12^, CH_4_、CO和SF_6_均能达到WMO/GAW实验室间比对分析拓展目标,能够很好地满足本底站高精度监测要求。

**表6:** 准确度测试结果

Analyte	Regression equation	R^2^	Calculated value	Calibrated value	Difference
CH_4_	y=0.023x-0.077	0.9999	2232.65×10^-9^	2236.8×10^-9^	0.15×10^-9^
CO	y=0.007x-0.152	0.9998	256.0×10^-9^	255.8×10-^9^	0.20×10^-9^
CO_2_	y=-0.002x^2^+41.202x-321.621	0.9999	453.75×10^-6^	453.38×10^-6^	0.37×10^-6^
N_2_O	y=0.023x^2^+11.798x+3622.442	0.9999	332.34×10^-9^	332.69×10^-9^	0.35×10^-9^
SF_6_	y=1.992x+1.066	0.9999	10.88×10^-12^	10.86×10^-12^	0.02×10^-12^

*y*: peak height (CH_4_, CO, SF_6_) or peak area (CO_2_, N_2_O); *x*: mole fraction.

### 2.4 试运行测试

图4为杭州市区2021年5~7月大气中60天CH_4_、CO、CO_2_、N_2_O、SF_6_的日变化特征。在城市地区,高强度的人为活动排放和气象条件是影响温室气体摩尔分数时空分布的主要因素^[22]^。如图4,在5~7月,除了SF_6_,大气CH_4_、CO、N_2_O和CO_2_均在下午14∶00~16∶00间达到最小值,且出现明显的早晚高峰现象。研究表明,夏季下午14∶00时分对流层边界层高度较高,大气对流活动强烈,垂直混合增强,有利于低层大气中气体的稀释和扩散^[23]^,同时CO_2_的低值又与日出后至下午的光合作用持续吸收CO_2_有关^[24]^。监测到的早晚峰值现象与城市上下班高峰时间对应,峰值主要来自于城市交通源的贡献^[25,26]^。CO_2_日变化呈现典型的单峰形态,日振幅为23.86×10^-6^,日平均摩尔分数为444.2×10^-6^±1.2×10^-6^,明显高于《中国温室气体公报》报道的杭州临安大气区域本底站2019年监测到的CO_2_摩尔分数(426.2×10^-6^±0.4×10^-6^),反映了杭州市区高度密集的交通源和工业源等强人为排放对大气CO_2_浓度的直接影响^[27]^。

**图4:**
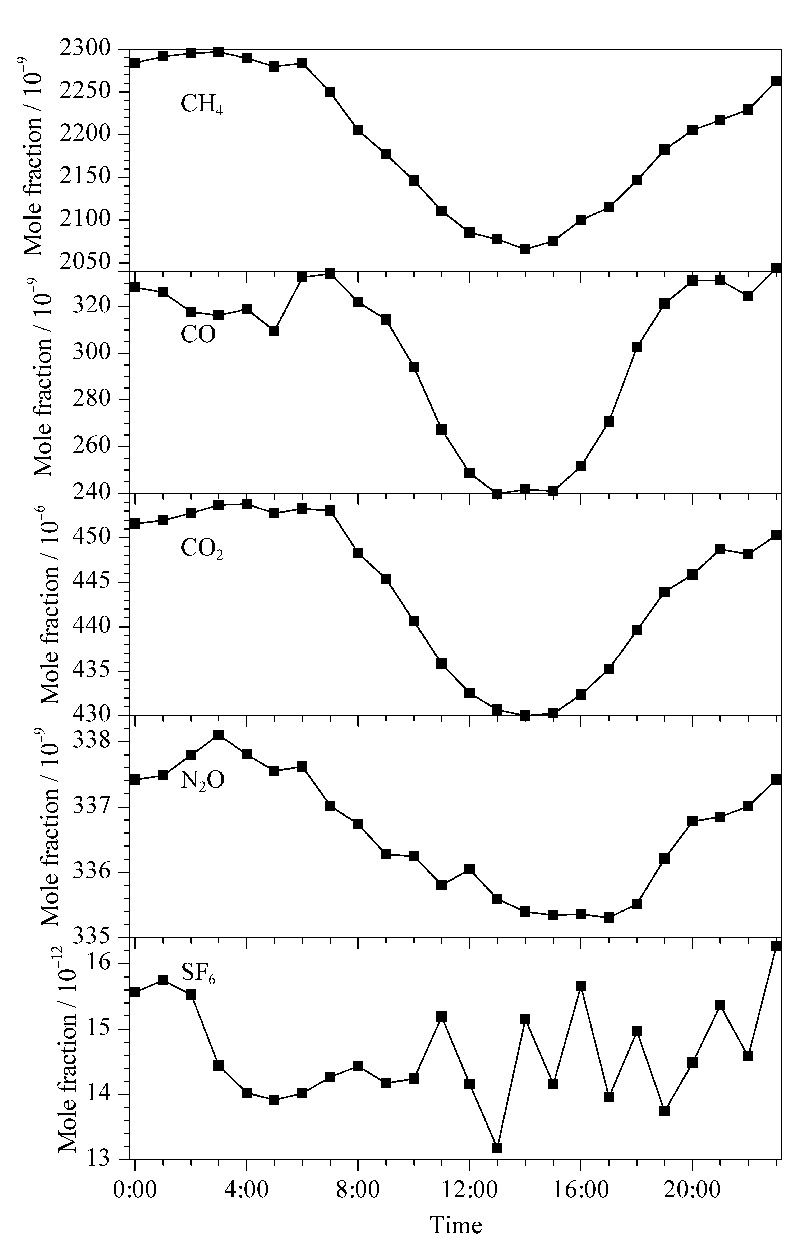
杭州市区2021年5~7月大气中的CH_4_、CO、 CO_2_、N_2_O、SF_6_摩尔分数日变化(*n*=60)

图5是2021年5~7月监测的杭州市区空气中CH_4_、CO、CO_2_、N_2_O和SF_6_含量的时间序列变化情况。其中CH_4_、CO、N_2_O和CO_2_在5月底即春末摩尔分数较高,到7月下旬有明显的下降趋势。在夏季,CH_4_、CO和CO_2_在大气中的汇增强,即CH_4_、CO与OH自由基活跃的光化学反应和陆地生态系统植物增强的光合作用对CO_2_的吸收。同时夏季边界层高于春季,有利于大气的混合与扩散。这种季节变化与北京上甸子、浙江临安、青海瓦里关等站点变化趋势一致^[28,29]^。目标气T的CH_4_、CO、CO_2_、N_2_O和SF_6_摩尔分数平均值分别为2072.2×10^-9^、189.0×10^-9^、414.15×10^-6^、319.89×10^-9^和10.25×10^-12^, SD分别为1.2×10^-9^、1.2×10^-9^、0.31×10^-6^、0.43×10^-9^和0.33×10^-12^, RSD分别为0.06%、0.65%、0.07%、0.13%和3.2%。T的响应结果显示系统在试运行期间精密度较好,CH_4_、CO符合WMO/GAW实验室间比对分析的拓展目标,完全能够应用于背景地区的浓度高精度监测。

**图5:**
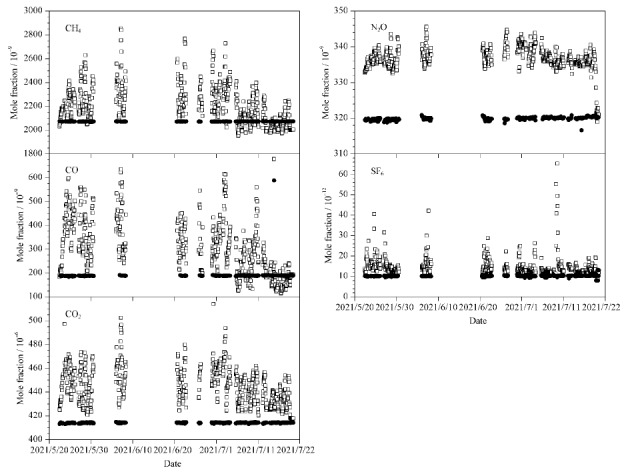
系统试运行期间杭州市区大气的CH_4_、CO、CO_2_、N_2_O、SF_6_摩尔分数变化情况

## 3 结论

本研究以传统气相色谱分析法为基础,通过自主设计、集成和优化调试,建立了高精度、高灵敏度、高准确度,且适用于环境大气中CH_4_、CO、CO_2_、N_2_O和SF_6_同步自动化连续监测的三通道气相色谱系统。该研发系统具有技术自主可控、运行成本更低、自动化水平更高等优势,能够满足城市温室气体高精度监测要求,可作为目前高精度温室气体监测仪器市场的重要补充。
